# The value of transbronchial lung biopsy in the diagnosis of lymphangioleiomyomatosis

**DOI:** 10.1186/s12890-021-01518-2

**Published:** 2021-05-03

**Authors:** Wenshuai Xu, Han Cui, Hongrui Liu, Ruie Feng, Xinlun Tian, Yanli Yang, Kai-Feng Xu

**Affiliations:** 1 Department of Pulmonary and Critical Care Medicine, State Key Laboratory of Complex Severe and Rare Diseases, Peking Union Medical College Hospital, Chinese Academy of Medical Sciences, Peking Union Medical College, Beijing, 100730 China; 2 Department of Pathophysiology, State Key Laboratory of Medical Molecular Biology, Institute of Basic Medical Sciences, Chinese Academy of Medical Sciences, Peking Union Medical College, Beijing, China; 3 Department of Pathology, Peking Union Medical College Hospital, Chinese Academy of Medical Sciences, Peking Union Medical College, Beijing, China

**Keywords:** Lymphangioleiomyomatosis, Biopsy, Transbronchial lung biopsy, Pathology, Diagnosis

## Abstract

**Background:**

Transbronchial lung biopsy (TBLB) in the diagnosis of lymphangioleiomyomatosis (LAM) is not a common approach, although TBLB is often performed in diffuse lung diseases. We aimed to examine the diagnostic value and safety of TBLB in LAM patients based on the data collected in our center.

**Methods:**

We reviewed LAM patients registered in our LAM Clinic from December 8, 2006, to December 31, 2019. All patients with definite or probable diagnosis of LAM who had been examined using TBLB were included. All available pathology slides were reviewed by an experienced LAM pathologist. All complications were reviewed by the medical records and confirmed using telephone interviews.

**Results:**

The pathology results of 86 patients (including 74 definite LAM and 12 probable LAM) were available. The positive rate of TBLB in LAM patients was 49/86 (57.0%). The positive rates of SMA, HMB-45, ER, and PR in LAM patients were 97.6%, 93%, 84.6%, and 78.4% respectively. The positive rate of TBLB was 40%, 60% and 60.8% in patients with CT Grade I, Grade II, and Grade III respectively, and the difference was not significant. Patients who had 3–4 or 5–6 biopsied specimens had a higher rate of diagnosis than those with 1–2 biopsied specimens. Four patients (5.6%) reported pneumothorax. No major hemoptysis was reported.

**Conclusions:**

TBLB is a feasible and safe procedure for obtaining a pathological diagnosis of LAM. Taking more than 2 samples during the biopsy procedure increased the rate of diagnosis.

## Introduction

Lymphangioleiomyomatosis (LAM) is a rare disease that predominantly affects females with clinical features of progressive cystic destruction of the lungs and the accumulation of LAM cells within the lungs and axial lymphatics [1]. Sporadic LAM affects 1 in 400,000 adult females, while it is common in adult females with tuberous sclerosis complex (TSC) [2]. The diagnostic criteria of definite LAM include characteristic or compatible features on high resolution CT of the chest and lung biopsy fitting the pathological criteria for LAM or characteristic high resolution computed tomography (HRCT)features and any of the following: renal angiomyolipoma, thoracic or abdominal chylous effusion, lymphangioleiomyoma or lymph node involvement by LAM, TSC or elevation of vascular endothelial growth factor-D (VEGF-D) in serum [2, 3]. In addition, there are many conditions that can mimic lung cysts and cause cystic lung disease, such as Langerhans cell histiocytosis, lymphoid interstitial pneumonia, and Birt-Hogg-Dubé syndrome [4]. Lung biopsy is important for the diagnosis of LAM. Transbronchial lung biopsy (TBLB) is less invasive than surgical lung biopsy. Current guidelines for LAM recommend TBLB before surgical lung biopsy [3]. However, the experience of this application has seldom been reported since the publication of this recommendation [5, 6]. We aimed to report our experience using TBLB on patients at the LAM Clinic in Peking Union Medical College Hospital (PUMCH), Beijing, China.

## Materials and methods

We reviewed LAM patients registered in the LAM center in PUMCH from December 8, 2006, to December 31, 2019, in our database to determine which patients had undergone TBLB. All patients with a definite diagnosis of LAM or probable diagnosis of LAM who had been examined by TBLB were included. The diagnosis criteria of definite LAM were based on ATS/JRS guidelines [3]. The diagnosis criteria of probable LAM were based on ERS guidelines [2]. The following data were collected: [1] diagnosis-related information: history of chylous effusion, HRCT of the chest, CT or magnetic resonance imaging (MRI) or ultrasound of abdomen, and serum VEGF-D; [2] number of samples biopsied during bronchoscopy; [3] pathological results including immunohistochemical examination; and [4] complications during bronchoscopy or within 24 h after the procedure.

Analyses of chest CT scans in these patients were conducted with digital CT images. The thickness of CT slices in most of the patients was high-resolution CT (HRCT), or no more than 5 mm. The severity of abnormalities on CT was graded according to the area of cysts involved: Grade I, cysts less than one-third of lung parenchyma; Grade II, cysts area between one-third and two-thirds of lung parenchyma; and Grade III, two-thirds or more [7].

All pathology slides were reviewed by experienced pathologists in LAM. Pathology slides from other hospitals were re-examined by one of our pathologists if the slides were available. Positive TBLB was defined as lung biopsies positive for HMB-45 or negative for HMB-45 biopsy showing typical LAM cell infiltration in hematoxylin and eosin-stained histological sections.

All complications were reviewed using medical records and confirmed using telephone interviews. Complication of moderate or severe hemoptysis was defined as hemoptysis that was persistent or required treatment. Pneumothorax was defined as a clinical manifestation of pneumothorax and confirmed by X-ray within 24 h of the procedure.

## Statistical method

Data were analyzed using SPSS for Windows version 24.0 (IBM Corp., USA) and are reported as the mean (SD). The *unpaired t-test* was used to compare continuous variables. Categorical variables were compared using *Pearson's chi-square test.* For all analyses, two-sided tests and a significance level of 0.05 were used.

## Results

### Patient information

**Fig. 1:**
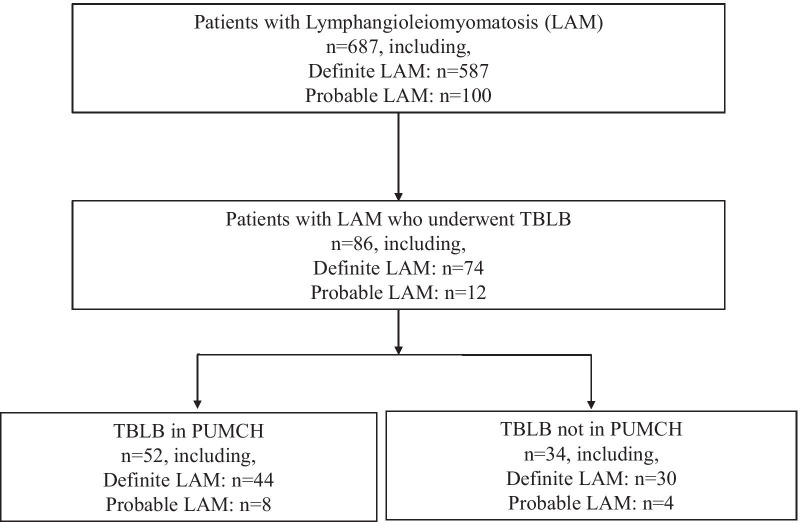
The flowchart of patient selection

Figure 1 shows the flowchart of the study. In detail, 687 LAM patients registered in PUMCH were reviewed, including 587 definite LAM patients and 100 probable LAM patients. The database recorded 86 of the 687 patients who had a history of TBLB, including 74 definite LAM patients and 12 probable LAM patients. Among 86 patients, 52 underwent TBLB in PUMCH, while 34 underwent TBLB in other hospitals. Six of 34 patients who underwent TBLB not in our hospital had pathological slides reviewed, 4 negative and 2 positive results. One of the 6 patients had the diagnosis revised from negative to positive after pathological slide review.

### Pathological diagnosis of LAM from TBLB samples

In total, the positive rate of TBLB for LAM diagnosis was 49/86 (57.0%). The positive rate of TBLB performed in PUMCH was 31/52 (59.6%). The positive rate of TBLB performed in other hospitals was 18/34 (52.9%).

**Fig. 2:**
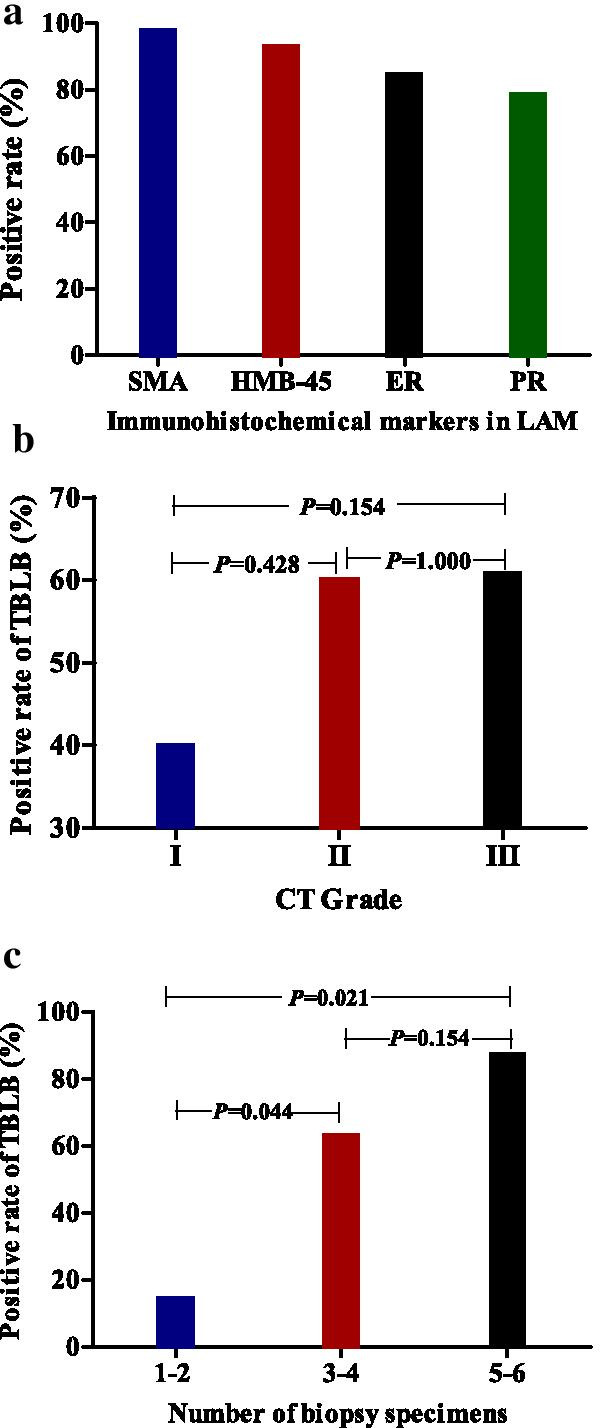
Pathological diagnosis of LAM from TBLB samples. **a** Positive rate of immunohistochemistry in positive TBLB patients. **b** Positive rate of TBLB in different CT grades of LAM patients. **c** Positive rate of TBLB in different numbers of lung biopsy specimens

For patients with negative TBLB, 20/37 (54%) patients did not have immunohistochemistry testing performed on their samples because there were no typical LAM cell infiltrations in hematoxylin and eosin-stained histological sections. Therefore, we only calculated the immunohistochemistry in the positive TBLB patients. The positive rate of smooth muscle actin (SMA) in patients with positive TBLB was 40/41 (97.6%). The positive rate of HMB-45 in patients with positive TBLB was 40/43 (93%). The positive yields of estrogen receptor (ER) and progesterone receptor (PR) were 33/39 (84.6%) and 29/37 (78.4%), respectively (Fig. 2a; Table 1).

**Table 1:** Characteristics comparison between positive TBLB and negative TBLB patients

Characteristics	Total TBLB N = 86	Positive TBLB N = 49	Negative TBLB N = 37	*P* value
Age, mean ± SD	42 ± 9	42 ± 9	41 ± 8	0.554*¶*
Definite LAM, N (%)	74 (86.0)	49 (100)	25 (67.6)	** < 0.001**
AML, N (%)	11 (12.8)	7 (14.3)	4 (10.8)	0.879
Chylothorax, N (%)	9 (10.5)	6 (12.2)	3 (8.1)	0.791
Chylous ascites, N (%)	1 (1.2)	1 (2.0)	0 (0)	1.000
Lymphangioleiomyoma, n/N (%)	26/77 (33.8)	16 (36.4)	10 (30.3)	0.574
VEGF-D > 800, n/N (%)	57/76 (75.0)	36/43 (83.7)	21/33 (63.6)	**0.045**
CT grade	n = 76	n = 43	n = 33	
I, N (%)	15 (19.7)	6 (14.0)	9 (27.3)	0.147
II, N (%)	10 (13.2)	6 (14.0)	4 (12.1)	1.000
III, N (%)	51 (67.1)	31 (72.0)	20 (60.6)	0.291
NO. of biopsy specimens	n = 61	n = 37	n = 24	
1–2 specimens, N (%)	7 (11.5)	1 (2.7)	6 (25.0)	**0.024**
3–4 specimens, N (%)	46 (75.4)	29 (78.4)	17 (70.8)	0.504
5–6 specimens, N (%)	8 (13.1)	7 (18.9)	1 (4.2)	0.201
*Immunohistochemical*				
SMA, n/N (%)	53/57 (93.0)	40/41 (97.6)	13/16 (81.25)	0.112
HMB-45, n/N (%)	40/60 (66.7)	40/43 (93.0)	0/17 (0)	** < 0.001**
ER, n/N (%)	38/50 (76)	33/39 (84.6)	5/11 (45.5)	**0.022**
PR, n/N (%)	34/48 (70.8)	29/37 (78.4)	5/11 (45.5)	0.083

Bold values indicate* P* < 0.05 was considered significant

AML: angiomyolipoma; CT: computed tomography; ER: estrogen receptor; PR: progesterone receptor; SMA: smooth muscle actin; TBLB: transbronchial lung biopsy; VEGF-D: vascular endothelial growth factor-D

The *P* value was calculated using *Pearson's chi-square test*

^*¶*^ The *P* value was calculated with the use of the *unpaired t-test*

### No difference in diagnostic yield in patients with different severity on CT scans

Seventy-six of 86 LAM patients had available CT scans for analysis. The positive rate of TBLB was 6/15 (40%) in Grade I LAM patients, 6/10 (60%) in Grade II LAM patients, and 31/51 (60.8%) in Grade IIILAM patients. However, there was no significant difference in the positive rate between Grade I, Grade II, and Grade III LAM patients (Grade I vs Grade II, *P* = 0.428; Grade I vs Grade III, *P* = 0.154; Grade II vs Grade III, *P* = 1.000) (Fig. 2b).

### Number of biopsy specimens influence the diagnostic yield

Sixty-one of the 86 reports recorded the number of biopsy specimens. Seven patients had 1–2 specimens taken, and the positive rate was only 1/7 (14.3%). Thirty-night patients were 3–4 specimens taken, and the positive rate was 29/46 (63.0%). Seven patients had 5–6 specimens taken, and the positive rate was 7/8 (87.5%). There were significant differences in positive rates between LAM patients with 1–2 specimens and patients with 3–4 specimens (*P* = 0.044). However, there was no significant difference between patients with 3–4 specimens and patients with 5–6 specimens (*P* = 0.154) (Fig. 2c).

### Complications of TBLB in LAM

Seventy-one of the 86 patients replied to the telephone questionnaire of the complications after the TBLB procedure. Four patients (5.6%) reported pneumothorax, including, three patients had 3 specimens taken, and one patient had 1 specimen taken. Additional biopsies of 5–6 specimens did not increase the rate of PTX. No major hemoptysis was reported. No other complications, such as pulmonary infections, were found.

## Discussion

Diagnostic yield was favorable for TBLB in the diagnosis of LAM with varying degrees of lung involvement. The major finding of this work is a 57% positive rate of diagnosis and 5.6% TBLB-related pneumothorax. The positive rate was different between patients who had 1–2 biopsied samples and 3 or more.

Pathology is essential for LAM patients who cannot be definitely diagnosed based on the clinical presentations, VEGF-D, and radiological appearance. In 1991, Pedreira et al. first reported that TBLB was used in the diagnosis of LAM [8]. In 1993, Bonetti et al. recommended that HMB45 should be used to distinguish LAM from other smooth muscle proliferation; the positive rate of HMB-45 in LAM was 92%, and the histological diagnosis of LAM could be made when only a transbronchial biopsy is available [9]. In 2012, Torre and Harari et al. reported that TBLB was performed in seven patients and was diagnostic in six and did not result in complications [10]. Meraj et al*.* found that TBLB was positive in 6 of 10 patients and had a yield of approximately 60% in LAM, with 52/217 (14%) complications, including pneumothorax (6%), bleeding (4%), chest pain (2%) and pneumonia (2%) [11]. In recent years, some researchers reported that the positive rate of TBLB in patients with LAM was 70–78%, and there were no serious adverse events such as pneumothorax or uncontrollable bleeding due to TBLB [5, 6]. Our results offered similar experience of the value of TBLB in LAM. The positive rate of TBLB in our results was similar to those previously reported [5, 6, 12]. In addition, the complications of TBLB were also similar to previous reports.

Immunostaining with HMB-45 antibody is considered to be the most reliable method for the specific identification of LAM cells [13]. In our study, the positive yield of HMB-45 in the diagnostic biopsies was 93%, which is similar to the previously reported rate of 92% [9]. HMB-45 was not positive in all LAM cells, but it was a valuable marker in LAM [14]. Gao et al.reported that in LAM, the expression of progesterone receptor is frequently higher than that of estrogen receptor [14].However, our pathological reports showed that the positive yields of estrogen receptor (ER) and progesterone receptor (PR) were quite similar (84% and 78%, respectively).

Severity of LAM on CT scans or pulmonary function test may influence the yield of TBLB. Koba et al*.* found the yield of TBLB was higher in patients with a reduced diffusing capacity [5]. Okamoto et al. reported the severity of LAM on CT scans affected the positive rate of TBLB [6].In our study Grade I LAM patients had a lower positive rate of TBLB than Grade II/III LAM patients although this was not statistically significant. The lack of statistical significance may have resulted from the small sample sizes in each group.

We found that with the increasing number of specimens, the positive rate will improve. The statistically significant increase in yield in going from 1–2 biopsy samples to 3–4 samples. While not statistically significant, there was yet another trend of increase in group of 5–6 samples. Small sample size probably precludes the results to be significant in the comparison of 3–4 samples and 5–6 samples. Increasing the number of specimens did not increase the risk of complications in our reports. However, there is a concern of potential of increased risk of complications when biopsy numbers increase. After balance the potential risk and benefit, we recommend that the number of samples biopsied could be 3–4 during the procedure.

Four patients (5.6%) reported pneumothorax, and no one reported major hemorrhage. Previous reports of TBLB in LAM found 0%-6% pneumothorax and 0%-4% hemorrhage [5, 6, 12].

De Fenoyl et al. reported that pneumothorax occurred in 3.4% of patients with interstitial lung disease who underwent TBLB [15]. The complication of pneumothorax in LAM may be slightly higher than in patients with interstitial lung disease. Evaluation before, during and after the procedure is critical in the early finding of pneumothorax. Large bulli should be avoided during the procedure.

TBLB is effective and safe for patients with LAM; however, some patients cannot be diagnosed with TBLB. Recently, transbronchial cryobiopsy (cryo-TBB) has been introduced as a technique to obtain biopsy samples of lung parenchyma that exceed the size and quality of forceps biopsy samples [16–18]. Cryo-TBB yields larger biopsy specimens (43 to 64 mm^2^) than transbronchial biopsy (5.8 mm^2^, range 0.58 to 20.88 mm^2^) [16, 19, 20]. Although crush artifact of TBLB for LAM diagnosis is not a major concern, cryo-TBB may increase the diagnostic yield not only because of increased sample sizes, but also the less injury of tissue structure biopsied. Cryo-TBB in patients with central, peribronchial or diffuse interstitial processes is now used clinically. The major complications of cryo-TBB included pneumothorax (12 to 28%), moderate bleeding (0 to 39%), and rarely death (0.3%) [17, 18, 20]. However, to date there have not been any large case series in LAM patients reporting on the utility of cryo-TBB for diagnosis. Pneumothorax risk is a major concern of cryo-TBB in LAM patients.

One point should be emphasized. Not all patients require pathological evidence for diagnosis. The definite diagnosis of LAM can be made based on characteristic presentations (chylothorax, renal angiomyolipoma, or tuberous sclerosis complex, etc.) or elevation of VEGF-D if characteristic cystic changes in lungs exist. Familiar with clinical features of LAM and tuberous sclerosis complex is essential for appropriate diagnosis. If pathological diagnosis is required, TBLB is recommended first before surgical lung biopsy [3].

There are some limitations to this study. First, it involved a small group of Grade I and Grade II patients; therefore, we do not clearly know whether the severity of CT grades will influence the positive rate of TBLB. Second, how to evaluate the risk of pneumothorax or hemorrhage before the TBLB procedure has not been investigated.

## Conclusion

We concluded that TBLB was a favorable and safe diagnostic procedure for LAM. An increasing number of specimens may improve the positive rate. The complication of pneumothorax was low. We support the ATS/JRS guidelines that recommended TBLB should be considered before surgical lung biopsy is considered.

## Data Availability

The dataset used and analyzed in current study available from the corresponding author.

## Abbreviations

CT: Computed tomography
ER: Estrogen receptor
PR: Progesterone receptor
SMA: Smooth muscle actin
TBLB: Transbronchial lung biopsy
